# Supplementary material for: Development and validation of the Iranian Minimum Data Set for Epidermolysis Bullosa: A mixed method approach
Source: PLoS One. 2025 Jan 7;20(1):e0316791. doi: 10.1371/journal.pone.0316791 (PMC11706488; doi:10.1371/journal.pone.0316791)
Supplement: S2 Table — (DOCX) [file pone.0316791.s002.docx]

| **Subclass** | **Data element** | **Frequency*** | **Percentage (%)** |
| --- | --- | --- | --- |
| Demographic data | national code | 25 | 100 |
|  | gender | 25 | 100 |
|  | date of birth | 25 | 100 |
|  | ethnicity | 24 | 96 |
|  | race | 24 | 96 |
| Address data of residence | province | 25 | 100 |
|  | city | 25 | 100 |
|  | village | 25 | 100 |
|  | full address of residence | 22 | 88 |
|  | telephone Number | 24 | 96 |
|  | mobile phone number | 25 | 100 |

**Table 1: Final Administrative Data Elements for EB (Frequency and Percentage)**

* The number of participations in Delphi Technique with "very important," "important" opinion

**Table 2: Final Clinical Data Elements for EB (Frequency and Percentage)**

| **Subclass** | **Data element** | | | | | **Frequency** | **Percentage (%)** |
| --- | --- | --- | --- | --- | --- | --- | --- |
| **Clinical symptoms** | Anatomical site involved | | | genitalia | | 25 | 100 |
|  |  |  |  | anus | | 25 | 100 |
|  |  |  |  | hand | | 25 | 100 |
|  |  |  |  | feet | | 25 | 100 |
|  |  |  |  | elbow | | 25 | 100 |
|  |  |  |  | knee | | 25 | 100 |
|  |  |  |  | lips | | 25 | 100 |
|  |  |  |  | face | | 25 | 100 |
|  |  |  |  | oral mucosa | | 25 | 100 |
|  |  |  |  | eyes | | 25 | 100 |
|  |  |  |  | neck | | 25 | 100 |
|  | Type of EB disease | | | simplex | | 25 | 100 |
|  |  |  |  | junctional | | 25 | 100 |
|  |  |  |  | dominant dystrophic | | 25 | 100 |
|  |  |  |  | recessive dystrophic | | 25 | 100 |
|  | Skin manifestations | | | blister | | 25 | 100 |
|  |  |  |  | erosion | | 25 | 100 |
|  |  |  |  | crust | | 25 | 100 |
|  |  |  |  | scar | | 25 | 100 |
|  |  |  |  | milia | | 25 | 100 |
|  |  |  |  | exuberant granulation tissue | | 25 | 100 |
|  | Disease severity | | | Localized | | 25 | 100 |
|  |  |  |  | Intermediate | | 24 | 96 |
|  |  |  |  | Severe | | 24 | 96 |
|  | Age of disease onset | | | | | 25 | 100 |
| **Complications caused by EB disease** | Eye | | corneal blister | | | 24 | 96 |
|  |  |  | corneal scar | | | 24 | 96 |
|  |  |  | corneal corrosion or abrasion | | | 25 | 100 |
|  |  |  | Symblepharon | | | 25 | 100 |
|  |  |  | blepharitis | | | 23 | 92 |
|  |  |  | ectropion | | | 22 | 88 |
|  |  |  | blockage of tear ducts | | | 23 | 92 |
|  |  |  | reduced vision | | | 25 | 100 |
|  |  |  | blindness | | | 25 | 100 |
|  | Gastrointestinal system | | esophagostenosis | | | 22 | 88 |
|  |  |  | abnormality or tooth decay | | | 24 | 96 |
|  |  |  | oral lesions | | | 25 | 100 |
|  |  |  | upper GI tract involvement | | | 25 | 100 |
|  | Genitourinary system | | ureteral stricture | | | 25 | 100 |
|  |  |  | urinary retention | | | 25 | 100 |
|  |  |  | bladder hypertrophy | | | 21 | 84 |
|  |  |  | hydronephrosis | | | 22 | 88 |
|  |  |  | ureteral stricture | | | 22 | 88 |
|  |  |  | pyelonephritis | | | 21 | 84 |
|  |  |  | cystitis | | | 23 | 92 |
|  |  |  | acute renal failure | | | 20 | 80 |
|  |  |  | constipation | | | 21 | 84 |
|  | Respiratory system | | upper respiratory system failure | | | 22 | 88 |
|  |  |  | lower respiratory system failure | | | 22 | 88 |
|  |  |  | nasal mucosa lesions | | | 21 | 84 |
|  |  |  | airway obstruction | | | 24 | 96 |
|  | Growth retardation | | | | | 22 | 88 |
|  | Anemia | | | | | 23 | 92 |
|  | Musculoskeletal system | | deformity of hands | | | 25 | 100 |
|  |  |  | deformity of legs | | | 25 | 100 |
|  | Nervous system (muscular dystrophy) | | | | | 22 | 88 |
|  | Skin complications | | alopecia | | | 25 | 100 |
|  |  |  | hair loss | | | 25 | 100 |
|  |  |  | nail dystrophy | | | 25 | 100 |
|  |  |  | milia | | | 25 | 100 |
|  |  |  | pigmentation | | | 25 | 100 |
|  | Carcinoma and neoplasms | | | | | 22 | 88 |
| **Diagnostic procedures** | Biopsy or sampling data | skin | | | | 25 | 100 |
|  |  | Chorionic Villus Sampling | | | | 25 | 100 |
|  |  | amniocentesis | | | | 25 | 100 |
|  | Molecular genetic testing | | | | | 25 | 100 |
| **Treatment procedures** | Complementary and alternative drugs | | | | iron | 23 | 92 |
|  |  |  |  |  | vitamin D | 22 | 88 |
|  | Medicines | | | | antibiotics | 25 | 100 |
|  |  |  |  |  | antidepressants | 23 | 92 |
|  |  |  |  |  | pain reliever | 25 | 100 |
|  |  |  |  |  | epilepsy drugs | 22 | 88 |
|  | Palliative procedures | | | | protective and non-adhesive dressings | 25 | 100 |
|  |  |  |  |  | draining blisters | 25 | 100 |
|  |  |  |  |  | reducing skin friction | 25 | 100 |
|  |  |  |  |  | keeping the skin cool | 25 | 100 |
|  | Rehabilitation procedures | | | | physiotherapy | 25 | 100 |
|  |  |  |  |  | ergotherapy | 25 | 100 |
|  |  |  |  |  | hydrotherapy | 23 | 92 |
|  |  |  |  |  | Psychotherapy | 25 | 100 |
| **Death data from EB disease** | Cause of death from EB disease | | | | death from sepsis | 25 | 100 |
|  |  |  |  |  | failure to thrive | 25 | 100 |
|  |  |  |  |  | airway obstruction | 25 | 100 |
|  |  |  |  |  | squamous cell carcinoma | 25 | 100 |
|  | Date of death | | | | | 25 | 100 |
| **Personal and family medical history data** | History of the person's disease | | | | underlying disease | 22 | 88 |
|  |  |  |  |  | underlying disease name | 22 | 88 |
|  |  |  |  |  | underlying disease duration | 22 | 88 |
|  |  |  |  |  | treatments provided | 25 | 100 |
|  | Family history of EB disease | | | | family relationship | 23 | 92 |
|  |  |  |  |  | type of disease | 23 | 92 |
|  |  |  |  |  | history of infection | 23 | 92 |
